# Free-Association Session Scale: factor structure and preliminary validity test

**DOI:** 10.3389/fpsyg.2023.1169372

**Published:** 2023-06-01

**Authors:** Rachele Mariani, Cinzia Di Monte, Luca Caricati, Tiziana Bastianini, Anna Ferruta, Christian Christopher, Anna Maria Speranza, Benedetta Guerrini degli Innocenti, Alessandro Musetti

**Affiliations:** ^1^Department of Dynamic and Clinical Psychology and Health Studies “Sapienza”, University of Rome, Rome, Italy; ^2^Department of Humanities, Social Sciences and Cultural Industries, University of Parma, Parma, Italy; ^3^Società Psicoanalitica Italiana, International Psychoanalytical Association, Rome, Italy; ^4^Department of Psychology, City College, City University of New York, New York, NY, United States

**Keywords:** psychoanalysis, free-association, psychotherapy process, clinician measure, quality session

## Abstract

One of the main concepts of the psychoanalytic method postulated by Freud in 1912 is the fundamental rule, which involves asking the patient to say whatever comes to mind as the analyst follows the patient's speech with fluctuating attention. Despite different theoretical models, this concept has remained an invariant element that characterizes the psychoanalytic method. For this reason, the purpose of the current study is to present a new instrument that measures this process based on the clinician's assessment. The Free-Association Session Scale (FASS) has been designed according to the psychoanalytic framework. Study 1 presented the preliminary validation of the FASS factor structure. Experienced Italian psychoanalysts (*N* = 281; 196 women) completed the FASS and sociodemographic questionnaire. The following two factors were identified using exploratory factor analysis: (1) Perturbing, and (2) Associativity. Study 2 cross-validated the two factors using an independent sample (*N* = 259; 187 women) of experienced psychoanalysts and confirmatory factor analysis (CFA). The FASS has been tested for concurrent validity using the Session Evaluation Questionnaire (SEQ) and Linguistic measures of the Referential process. The two-factor model achieved a close-fit test, and the FASS items were found to measure the corresponding factors with good reliability. The Perturbing factor is negatively associated with three SEQ factors (Depth, Smoothness, and Positivity) and negatively correlated with symbolization (IWRAD and IWRAD_IWRRL), confirming a more complex and unexpected session. The Associativity factor is positively associated with all four SEQ factors (Depth, Smoothness, Positivity, and Arousal). In conclusion, the FASS is a promising new questionnaire for assessing psychoanalytic session quality processes with satisfactory validity and reliability.

## 1. Introduction

The *fundamental rule of psychoanalysis* (Freud, 1912)—that a patient should say whatever comes to mind—indicates free associations, which Kris (1956) believed was “the hallmark of psychoanalytic treatment conducted by analysts of every stripe” (p. 26). The concept of free association is considered a fundamental process, defining the work carried out in psychoanalysis, by which patients and analysts are discouraged from imposing any censorship on the flow of their thoughts and associations. In his recommendations for practicing psychoanalysis, Freud wrote, “Just as the patient must relate everything that his self-observation can detect, and keep back all the logical and affective objections that seek to induce him to make a selection from among them, so the doctor must put himself in a position to make use of everything he is told for the purposes of interpretation and of recognizing the concealed unconscious material without substituting a censorship of his own for the selection that the patient has forgone. To put it in a formula: he must turn his own unconscious like a receptive organ toward the transmitting unconscious of the patient. He must adjust himself to the patient as a telephone receiver is adjusted to the transmitting microphone. Just as the receiver converts back into sound waves the electric oscillations in the telephone lines which were set up by sound waves, so the doctor's unconscious is able, from the derivatives of the unconscious which are communicated to him, to reconstruct that unconscious, which has determined the analysand's free-associations” (p. 115–116).

Freud attempted to convey the fundamental rule in the treatment by using his now-famous metaphor of the patient acting like a passenger on a train, whereby the patient should report on their spontaneously arising thoughts as a passenger on a train might report on the views they see out of their window: “Act as though, for instance, you were a traveler sitting next to the window of a railway carriage and describing to someone inside the carriage the changing views which you see outside” (p. 135).

What Kris described as the good hour is the patient's capacity for free associations. Kris wrote, “Many a time the ‘good hour' does not start propitiously. It may come gradually into its own, say after the first ten or fifteen minutes, when some recent experience has been recounted, which may or may not refer to yesterday's session. Then, a dream may come, and associations and all begins[sic] to make sense. In particularly fortunate instances, a memory from the near or distant past, or, suddenly, one from the dark days, may present itself with varying degrees of affective charge” (p. 446). Just as the patient is instructed to say whatever comes to mind, the analyst is also recommended to allow themselves to express their thoughts and reactions without censorship. The corollary to the fundamental rule for the patient is the recommendation of free associations to be conducted by the analyst through evenly hovering attention. In his oft-quoted metaphor of the phone receiver, Freud (1912) asserted that, if the analyst had no resistance of his own to the analysand's material, he would be able to reconstruct aspects of the patient's unconscious as communicated in his free associations. In other words, attention to the patient's flow and interruption of associations help the analyst identify and discuss defensive mechanisms. The idea of analytic neutrality linked to the decoding of free associations is one of the themes that contemporary psychoanalysis has reformulated and generated several controversies. In fact, many contemporary authors have extended the concept of free associations and given fluctuating attention to these associations with new definitions, concepts, and phenomena that we have included in our study to explicate the construct under consideration.

Many authors have debated the important technical role of free speech and the dysfunction of the fundamental rule (Kris, 1983, 1990; Adler and Bachant, 1996; Hoffer, 2006). In fact, the debate places, at the center of the operation of the two phenomena, central aspects of the analytic method, the transferential relationship, the countertransferential dimension, and the neutrality position conceived in contemporary terms (i.e., awareness of the analyst's role in subjective influence) (Parsons, 2006; Barratt, 2021).

In the contemporary view, the concepts of associative freedom and free-floating attention express not only parallel work but also mutually coactivating phenomena in a game of reciprocal tensions and pleasure-taking, oscillating between the enjoyment of enunciation and the risk of judgment and between the regressive drive and the elaborative/integrative drive. This broader conceptualization of free association as anything that is set in motion in the analysis room initially starts with the Kleinian conceptualization. As defined by Klein, the play of children was assimilated into free associations and dreams of adults. In fact, we included other definitions of free association related to more contemporary authors who highlighted specific definitions of these processes, such as Winnicott (2005) in Playing and Reality; Bion (1962) and his dimensions of PS ↔ D oscillation and the concept of reverie shifting into analytical free-floating attention (Bion, 1962); the definition of Weiss (1960) concerning the human ability “to perceive through its sense organs,” such as the ability to recognize through resonance and duplication in one's mind; the concept of “listening with the third ear” (Reik, 1983); the concept of unconscious communication (Loewald, 1960; Abend, 2018); the definition of the Central Phobic Position (Green, 2018); Dimensions of Embodied Communication (De Toffoli, 1991; Bucci, 2011); the formulation of unthought known (Bollas, 1987; Arizmendi, 2008); and unprocessed unconscious thoughts (Ogden, 2022). All these conceptualizations were examined and debated in the research group to come up with the specific descriptors of the constructs “free association” and “free-floating attention.” After several meetings, the group then elaborated on the clinical discussions and discussed the items that represented the most classic and contemporary definitions of these processes of functioning in the analysis room.

### 1.1. Psychotherapy research and clinicians' report instruments

Many studies have investigated the importance of clinical outcomes and process assessment tools evaluated by the clinician himself or herself. The importance and reliability of the clinician's assessment are fundamental in the study of psychotherapy, from diagnostic assessment (Wardenaar and de Jonge, 2013; Allsopp et al., 2019; ) to process and outcome evaluation (Bugatti and Boswell, 2022). In fact, many studies have pointed out that clinician-reported information is different from patient self-report assessments and can provide more information on the progress of clinical cases (Cuijpers et al., 2010). In the most recent studies on the use of clinician perception-based measures, while there is a tendency to overestimate the positive impact of treatment by clinicians (Krägeloh et al., 2015; Gondek et al., 2016), there is increasing evidence of a clinician's preference to use idiographic and more customizable process and outcome measures (Jensen-Doss et al., 2018). One of the first constructs that was investigated based not only on patients' self-reports but also on the therapist's assessment was the therapeutic alliance (Elvins and Green, 2008; Thompson and McCabe, 2012). This measure then spread to other instruments, whose versions were built for patients and therapists. Currently, many of the instruments have originated only through clinician reports and are more related to theoretical concepts such as transference, countertransference, and personality assessment (Tanzilli et al., 2016, 2018; Colli et al., 2019). Our instrument falls within this category because it is a clinician's report on the following two of the most significant theoretical concepts: free associations and free-floating attention.

Clinician report instruments have completely changed the perspective of the investigation of empirical research in psychotherapy (Cuijpers et al., 2010), attempting to accommodate the clinician's need to make it more tailored to the patient and the relationship between the two participants. This new orientation allowed the development of many widely used tools of psychodynamic optics, such as the Psychodynamic Diagnostic Manual Version (Lingiardi and McWilliams, 2017), the Shedler–Westen Assessment Procedure-200 (Shedler and Westen, 2007), transference and countertransference analysis, and defenses using the analyst's optics. Concurrent with the emergence of clinician-assessed instruments for diagnostic purposes and for the verification of intervention outcomes, an original piece of research has been developed on the use of one of the most widely used clinical tools since the origin of psychoanalysis, namely, clinician's reports. Bucci and Hoffmann's research is distinct because of their application method of linguistic analysis not to the transcribed sessions but to the clinician's notes (Bucci et al., 2012; Hoffman et al., 2013; Mariani and Hoffman, 2021). The original application of this method made it possible to create a link between the trend of emotional communication circles identified by Bucci (2021) in the session and the clinician's notes.

### 1.2. The present study

The present study aimed at examining the psychometric properties of a new self-report tool, called the Free-Association Session Scale (FASS), for clinicians to assess free-associative and free-floating attention functioning as defined above. To the best of our knowledge, this is the first instrument related to the psychoanalytic concepts of free association and free-floating attention. The FASS has been developed as a clinician's post-session scale to measure the quality of the psychoanalytic functioning between the analyst and the patient. Through focus groups, 36 descriptor items have been produced. The following two main areas were identified: (a) Descriptors of free-association and free-floating attention closer to the classical theoretical model (Freud, 1912, 1915; Kris, 1983) (*examples: item 9—the patient introduces something completely new to the analyst and item 10—the analyst is immersed in a stream of thoughts and a word from the patient catches their attention*); (b) descriptors of new definitions of free associations and fluctuating attention that take life from the bipersonal conceptualizations of contemporary psychoanalysis *(examples: item 1—the patient startles and says, “There was something I wanted to tell you” and item 21—the analyst becomes agitated and/or a symptom occurs in the session as palpitations, nausea, vomiting, fainting, headache, tic, ough ...)*.”

Thus, we carried out two studies. Study 1 used an exploratory factor analysis approach to assess the factor structure of the FASS. Study 2 used confirmatory factor analysis to validate hypotheses concerning the FASS factors and compared FASS with other measures already validated for concurrent validity. Accordingly, we explored the relationships between FASS and the evaluation of sessions regarding bad or good perceptions by analysts through the Session Evaluation Questionnaire (SEQ). Moreover, we explored the relationship between the FASS factors and the linguistic measures of the referential process applied to the analysts' session notes. Thus, the clinical notes that the analysts write after each session were analyzed, and the notes that referred to that session were evaluated using the FASS.

## 2. STUDY 1: exploratory factor analysis

### 2.1. Methods

#### 2.1.1. Participants and procedure

Psychoanalysts who were members of the Italian Psychoanalytic Society and the International Psychoanalytical Association for at least 5 years were recruited through snowball sampling. After providing online consent, participants were asked to fill out the instruments using an online form. The final sample consisted of 281 psychoanalysts (85 men and 196 women). Participants' mean age was 62.49 years (SD = 8.9, range 36–79), and the average length of clinical experience as a psychoanalyst was 16.01 years (SD = 13.89, age range 6–36). The psychoanalysts were requested to fill out the questionnaire considering the last session with their patients in treatment. The average length of treatment was 32.21 months (SD = 37.1, range 0–436), and the duration of weekly sessions ranged from 1 to 4 days. The participants were asked to assess the first patient of the day without specific criteria of selection because there were no patient exclusion criteria. This study had ethical approval by the Ethics Committee of the Department of Clinical, Dynamic Psychology, and Health Studies (Prot. n. 0001104 del 16/07/2021).

#### 2.1.2. Measures

Sociodemographic questionnaire. An ad hoc questionnaire was developed to collect the following data: gender and age of the psychoanalysts and their patients, years of experience as psychoanalysts, the number of sessions per week, months of treatment, and treatment context (vis-à-vis, the couch, online, telephone, or in person treatment), due to the study starting during the COVID-19 pandemic period.

Free-Association and Free Floating-Attention Scale (FASS; see Table 1). In its final form, the questionnaire was a 36-item clinician report that was to be filled out by the psychotherapists at the end of the session. The instructions request the psychotherapist to go over the entire session in his or her mind and indicate how salient that item is, representative of that specific session, where 1 indicates “not at all” and 4 indicates “extremely” significant. The following two specific areas were explored: the patient's free-associative functioning and the therapist's fluctuating attention.

**Table 1:** English and Italian final version of the Free-Association and Free Floating-Attention Scale (FASS).

Instruction: Dear Collegue,				
Concentrate on the session that has just ended and try to respond to the questions below by describng the session as a whole, considering the main impressions you took away from it. Keep in mind that 1 indicates not at all significant and 4 extremely significant.				
Istruzioni: Gentile Collega,				
Si concentri sulla seduta appena terminata e cerchi di rispondere alle affermazioni descrivendo la seduta nella sua globalità, rispetto a ciò che le è rimasto più impresso della seduta. Tenendo conto che 1 indica per nulla significativo 4 estremamente significativo.				
1. The patient startles and says: “There was something I wanted to tell you”/Il paziente sussulta: “ecco cosa volevo dirle”	1	2	3	4
2. The analyst says a word that is misunderstood by the patient from which, however, the patient continues to elaborate/L'analista dice una parola che viene fraintesa dal paziente dalla quale inizia però a parlare	1	2	3	4
3. Patient says: “I can't think of anything”/Il paziente dice: “non mi viene in mente nulla”	1	2	3	4
4. The patient says: “I wrote down the dreams I wanted to tell you about”/Il paziente dice: “mi sono scritto i sogni di cui volevo parlarle”	1	2	3	4
5. The analyst interrupts the patient's speech chain/L'analista interrompe la catena del discorso del paziente	1	2	3	4
6. An image or personal memory occupies the analyst's attention/All'analista viene in mente un'immagine o un ricordo che cattura la sua attenzione	1	2	3	4
7. The patient does not speak/Il paziente non parla	1	2	3	4
8. The analyst is bored, thinks about personal facts, and does not feel that these thoughts are useful/L'analista si annoia, pensa a fatti personali, e non sente utili questi pensieri	1	2	3	4
9. Patient introduces something completely new to the analyst/Il paziente dice una cosa del tutto nuova o che sa di nuovo	1	2	3	4
10. The analyst is immersed in a stream of thoughts and a word from the patient catches their attention/L'analista è immerso in un flusso di pensieri e una parola del paziente cattura la sua attenzione	1	2	3	4
11. At the end of the analyst's intervention, the patient says: “What you just said made me remember something.”/Al termine dell'intervento dell'analista, il paziente dice: “quello che ha appena detto mi ha fatto ricordare una cosa”	1	2	3	4
12. After a long silence, the patient starts talking/Dopo un lungo silenzio il paziente inizia a parlare	1	2	3	4
13. The analyst has the impulse to correct the patient because they said something wrong/All'analista viene da correggere il paziente perché ha detto una cosa sbagliata	1	2	3	4
14. The patient speaks in an intellectualized mode/Il paziente parla in una modalità intellettualizzata	1	2	3	4
15. The patient talks as if everything was already defined or “pre-packaged.”/Il paziente parla come se tutto fosse già definito e preconfezionato	1	2	3	4
16. The analyst struggles not to simply re-state what the patient is saying/L'analista fa fatica a non replicare	1	2	3	4
17. The analyst makes very theoretical interventions/L'analista fa delle comunicazioni molto teoriche	1	2	3	4
18. The analyst offers explanations/L'analista fornisce delle spiegazioni	1	2	3	4
19. A noise comes from outside and the patient does not comment/Un rumore proviene dall'esterno e il paziente non commenta	1	2	3	4
20. A noise comes from outside and the analyst comments/Un rumore viene dall'esterno e l'analista commenta	1	2	3	4
21. The analyst becomes agitated and/or a symptom occurs in the session (e.g. palpitations, nausea, vomiting, fainting, headache, tic, ough ...)/L'analista si agita e/o avviene un sintomo nella stanza (ad es. palpitazioni, nausea, vomito, sviene, mal di testa, tic, tosse...)	1	2	3	4
22. The analyst makes a slip of tongue (lapsus)/L'analista fa un lapsus	1	2	3	4
23. The analyst has a misperception that disorients them (stench, perfume, perception of something)/L'analista ha una dispercezione che lo disorienta (puzza, profumo, percezione di qualche cosa)	1	2	3	4
24. Patient says something that the analyst was also thinking right at the moment/Il paziente dice qualcosa che l'analista stava pensando proprio in quel momento	1	2	3	4
25. The analyst experiences a sensation that personally frightens them, distracts them and leads them to fidget/L'analista prova una sensazione che lo spaventa personalmente, lo distoglie e lo porta ad agitarsi	1	2	3	4
26. The analyst allows themself to be carried away by the patient's associations/L'analista si lascia portare via con il pensiero dalle associazioni del paziente	1	2	3	4
27. The patient relates episodes or memories and comes to a new understanding about themself/Il paziente racconta degli episodi o dei ricordi e capisce delle cose nuove di sé	1	2	3	4
28. The patient responds to the analyst's interpretation by recalling a story, a dream, or a memory/Il paziente risponde all'interpretazione dell'analista collegando un racconto o un sogno o un ricordo	1	2	3	4

To obtain Factor 1- Perturbing-, add the following items: 1; 2; 3; 4; 5; 7; 8; 12; 13; 14; 15; 16; 17; 18; 19; 20; 21; 22; 23; 25. Mean score in this study is 35.11 SD = 1.67.

To obtain Factor 2-Associativity-, add the following items: 6; 9; 10; 11; 24; 26; 27; 28. Mean score in this study is 21.62 SD = 4.13.

#### 2.1.3. Statistical analyses

Exploratory factor analysis (EFA) was performed to investigate the expected two-factor structure of FASS. Given that it has been shown that treating items with less than 4 response categories might produce biased results (Johnson and Creech, 1983; Zumbo and Zimmerman, 1993), we considered responses to be ordinal. Accordingly, EFA was performed based on polychoric correlations among items and by considering maximum likelihood as the factor method with Varimax rotation. We retained items with loadings greater than 0.30 on only one factor, and items with loadings greater than 0.29 on two items were considered to have low discriminant validity, which were then removed from the scale.

### 2.2. Results

Exploratory factor analysis revealed that two factors explained more than 51% of the total variance of items. However, three items had loadings lower than 0.30 on both factors (items 8, 7, and 17), while three items had loadings >0.30 on both factors (items 41, 49, and 24). These items were then excluded, and a further EFA was performed, indicating that item 47 also has loadings greater than 0.30 on both factors. After excluding this item, EFA revealed that all remaining items had loadings >0.30 on one factor only (see Table 2) and that the two-factor solution explained 58% of the total variance. The first factor grouped items referring to the associative processes of the patient, and the fluctuating attention of the analyst were descriptors adhering to the most classic definition of psychoanalysis according to the Freudian model, named “Associativity.” The second factor grouped items referring to a more relational contemporary psychoanalytic conceptualization that highlights a more unpredictable emotional dynamic consistent with relational theory. It signals perturbations and aspects of novelty, named “Perturbing.” Cronbach's alpha indicated that reliability was good for both factors, as expressed by 0.95 and 0.73, respectively, and McDonald's Omega was expressed as 0.93 and 0.70, respectively.

**Table 2:** Results of exploratory factor analysis.

	**Study 1**		**Study 2**	
	**Factor 1**	**Factor 2**	**Factor 1**	**Factor 2**
It13	0.89		0.82	
It23	0.87		0.82	
It21	0.86		0.86	
It3	0.85		0.77	
It2	0.85		0.62	
It19	0.84		0.72	
It25	0.83		0.74	
It8	0.82		0.74	
It7	0.82		0.65	
It15	0.82		0.70	
It17	0.81		0.76	
It12	0.79		0.66	
It4	0.78		0.66	
It22	0.78		0.75	
It14	0.78		0.32	
It5	0.77		0.62	
It20	0.76		0.78	
It16	0.71		0.59	
It1	0.70		0.48	
It18	0.63		0.49	
It27		0.73		0.61
It28		0.67		0.73
It24		0.60		0.47
It11		0.58		0.61
It6		0.56		0.38
It9		0.53		0.45
It26		0.51		0.60
It10		0.46		0.40

## 3. STUDY 2: confirmatory factor analysis

### 3.1. Methods

#### 3.1.1. Participants and procedure

We recruited a sample of psychoanalysts using snowball sampling through email invitations. After receiving a brief presentation of the study, participants were asked, via Google Form, to complete the questionnaires and write a brief clinical overview of the session, which we will call clinical “notes” here. This type of account is widely used in the clinical practice of psychoanalysts to compare clinical and theoretical practice or for case supervision. This sample consists of 259 psychoanalysts (*M* age = 61.39 years; SD 8.9; range 36–79; 72 males and 187 females). The average length of participants' clinical experience as psychoanalysts was 13.78 years (SD = 9.76, range 6–36). Participants filled out the questionnaire considering the last session with their patients in treatment. The average length of treatment was 30.39 months (SD = 35.1, range 0–120), and the frequency of weekly sessions ranged from 1 to 4 days. The average patient's age is 37.90 years (SD = 12.4, range 10–82). Participants were free to choose any patient to refer to in order to complete the questionnaire; there were no patient exclusion criteria. The psychoanalysts had to have been members of the Italian Psychoanalytic Society for at least 5 years in order to be included. This study had ethical approval from the Ethics Committee.

#### 3.1.2. Measures

Sociodemographic questionnaire. This was carried out in the same manner as in Study 1. Moreover, because this data collection was carried out during the COVID-19 pandemic, several contexts of treatment were explored as follows: vis-à-vis, telephone, or video call.

Free-Association and Free Floating-Attention Scale (FASS). This is the same as in Study 1.

Session evaluation questionnaire (SEQ; Stiles, 2002). The SEQ, version 5, has 21 items in a 7-point bipolar adjective format. The following instructions are given to respondents: “Please circle the relevant number to reflect how you feel about this session.” The items are divided into two sections: session evaluation and post-session mood. SEQ consists of 4 scales: “Depth,” which assesses the session's perceived power and value (e.g., valuable-worthless and shallow-deep); “Smoothness,” which assesses the degree to which the session's atmosphere was perceived as comfortable, relaxed, and pleasant; “Positivity,” which assesses feelings of confidence and clarity, as well as happiness and the absence of fear or anger; and “Arousal,” which assesses feelings of being active and excited as opposed to feelings of quiet and calm.

Linguistic measures of the referential process. The Italian Discourse Attributes Analysis Program (IDAAP) was designed to read texts and compare them word by word. The IDAAP utilizes several dictionaries simultaneously. We used the following dictionaries for the Italian language in this study, based on psychoanalysts' notes:

Italian Weighted Referential Activity Dictionary (IWRAD): The computerized measure of RA is represented by the Italian Weighted Referential Activity Dictionary (IWRAD). High scores indicate high referential activity. The majority of words with low IWRAD weight scores have a subjective focus as opposed to referring to outside objects and describing circumstances in the present tense as opposed to the past tense.
The Italian Reflection Dictionary (IREF): This is a dictionary with 908 abstract terms that describe how individuals think and express their ideas. It contains terms from cognitive or logical thinking as well as basic logic.
The Italian Sensory Somatic Dictionary (ISensD): This is a dictionary with 1,926 terms that are connected to the body and physical functions. A measure of sub-symbolic activation is the number of ISensD words in a speech sample.
The Italian Sum Affect Dictionary (ISAffD): This is a dictionary with 1,786 terms describing how people feel and express their emotions [positive affect (IAffP), negative affect (IAffN), and neutral affect (IAffZ)].
The Italian Weighted Reflection and Reorganization List (IWRRL): This refers to the reorganization and reflection function, in which a speaker makes an effort to identify and comprehend the emotional significance of a particular event or series of related events. High results on this test, which includes a list of 1,633 Italian words, indicate high levels of reflection and reorganization.

#### 3.1.3. Statistical analyses

Preliminarily, an EFA (as done in Study 1) was performed on items to investigate whether the factor structure would be similar to that of Study 1. Next, a confirmatory factor analysis (CFA) was performed considering a two-factor structure. A weighted least squares mean and variance adjusted (WLSMV) estimator was used because it is suitable for taking the ordinal nature of items into account and provides robust parameter estimates and standard errors. We considered factor loadings to be excellent, very good, good, fair, and poor at values of 0.71, 0.63, 0.55, 0.45, and 0.32, respectively (Comrey and Lee, 2013). The fit of the model was evaluated considering the comparative fit index (CFI), the Tucker-Lewis index (TLI), and the root mean square error of approximation (RMSEA). For CFI and TLI, values greater than 0.90 and 0.95 were considered adequate and excellent (Marsh, 2007; Perry et al., 2015). For RMSEA, values lower than 0.08 and 0.06 were assumed to indicate adequate and excellent fit. We also reported the chi-squared test and the ratio between chi-squared and degrees of freedom. It is worth noting that the chi-squared value is inflated when large samples are considered, while the chi-squared/df ratio should not exceed 3. To test concurrent validity, Pearson correlations were performed between confirmed factors and sociodemographic variables, session evaluation quality (SEQ), and referential process (RP) language measures. As indicated in Table 2, factorial solutions from EFA in Study 1 and Study 2 were largely overlapping, indicating the relative robustness of the factor structure.

### 3.2. Results

#### 3.2.1. Confirmatory factor analysis

The results of CFA revealed that the fit of the two-factor model was adequate, χ^2^ (349) = 791.48, *p* < 0.001; χ^2^/df = 2.27, CFI = 0.92, TLI = 0.91, RMSEA = 0.07, 95% CI [0.06; 0.08], *p* < 0.001. Moreover, all items were significantly measured by the intended latent trait (all *p*-values < 0.001). To increase the fit of the model, we relaunched CFA by excluding items that indicate poor loading on latent traits, namely, items 13, 40, and 27. Analysis without these items yielded a better and good fit of the model, χ^2^(274) = 437.38, *p* < 0.001; χ^2^/df = 1.60, CFI = 0.96, TLI = 0.96, RMSEA = 0.05, 95% CI (0.05; 0.06), *p* = 0.244. *Post-hoc* power analysis revealed that the analyzed sample has a power greater than 90% to detect the misspecified model (as indicated by an RMSEA ≥ 0.05) with a level of alpha = 0.05.

#### 3.2.2 Relationship between FASS, sociodemographic, and setting-related variables

To explore the relationship between FASS factors and sociodemographic dimensions, a correlational analysis was carried out. The Perturbing factor (FASS) is related to the patient's age (*r* = −0.154; *p* = 0.014) and years of experience as psychoanalysts (*r* = −0.490; *p* < 0.001). The Associativity factor (FASS) showed a relationship with the psychoanalysts' age (*r* = −0.165; *p* = 0.010). The correlations between FASS and patient and analyst ages were not observed as clinically significant because we defined the validity r of >0.30 as significant.

A one-way ANOVA analysis was used to examine differences in FASS factor scores for some setting characteristics: setting (couch and *vis-à-vis*), sessions per week, and mode of the session (telephone, in presence, and video call). The results showed that there were no differences in FASS factors in relation to these dimensions.

#### 3.2.3. Correlational analysis between FASS, SEQ, and linguistic measures

As shown in Table 2, all factors of SEQ were significantly related to FASS factors. Moreover, the results of the correlational analysis between FASS factors and linguistic measures are reported in Table 3.

**Table 3:** Correlation analysis between FASS, SEQ, and linguistic measures.

	**Perturbing FASS**	**Associativity FASS**
Depth (SEQ)	−0.161^**^	0.353^**^
Smoothness (SEQ)	−0.249^**^	0.277^**^
Positivity (SEQ)	−0.472^**^	0.248^**^
Arousal (SEQ)	0.006	0.149^*^
Words	−0.080	−0.289^**^
MIWRAD	−0.210^*^	0.113
MIWRRL	0.104	0.058
MiRef	−0.166	0.038
MIAffN	0.069	0.019
MIAffP	0.032	0.081
MIAffZ	−0.192^*^	−0.106
IWRAD_IWRRL	−0.261^*^	0.169

^*^p < 0.05.

^**^p < 0.001.

MIWRAD, Mean Italian Weighted Referential Activity Dictionary; MIWRRL, Mean Italian Weighted Reflection and Reorganization List; MiRef, Mean Italian Reflection Dictionary; MIAffN, Means Italian Affect Negative; MIAffP, Means Italian Affect Positive; MIAffZ, Means Italian Affect Neutral; IWRAD_IWRRL, Italian Weighted Referential Activity Dictionary and Italian Weighted Reflection and Reorganization List.

## 4. Discussion

The present study aimed to test the psychometric properties of the new instrument for measuring free associations and fluctuating attention. This instrument is inspired by the psychoanalytic constructs of free association and free-floating attention, first defined by Freud in 1912. The results confirmed the initial hypothesis of the two factors. The CFA confirmed two defined factors: Associativity and Perturbing. These two factors revealed two aspects of free-associative functioning. The first is more consistent with a classical model of functioning in session, that is, Freud's theory of following one's flow of thoughts by both the patient and the analyst. The second factor describes free associative functioning as an element of an “event” happening at the moment. This can refer to something that is unexpected for both the patient and the analyst or something that disrupts the relationship between the patient and analyst and forces them to rethink the new happening. Many contemporary psychoanalytic theories (Bollas, 1987; Green, 2000, 2018; Ogden, 2022) emphasize that events and happenings in the clinical exchange are other dimensions of free associations. The results of the second study highlight that the model of interpreting clinical dimensions is not dependent on the setting used but is consistent with the analyst's theoretical training. This finding is important precisely because of the use of an instrument that captures the clinician's point of view.

The results regarding the relationships between demographic variables and FASS factors revealed that the Perturbing factor of FASS is negatively correlated with patients' age, while the Associativity factor of FASS is negatively correlated with psychoanalysts' age. This age-related result confirms that adolescent or young adult patients tend to use a less reflective Associative method; with younger patients, there is more action in the therapeutic setting and later acquisition of meaning. Thus, adult patients are more reflective and adhere more to the ground rule as described in the classical model. The inverse correlation between the age of psychoanalysts and the Associativity factor is a very interesting result that needs further investigation because it might highlight how younger psychoanalysts report greater adherence to the more classically defined free association rule, whereas more experienced psychoanalysts acquire greater freedom of the free associative process.

By relating the FASS factors to the SEQ factors, we found that Associativity is positively associated with all four SEQ factors (Depth, Smoothness, Positivity, and Arousal). This indicates a good session based on the psychoanalyst's experience. High Associativity scores consistent with the classic definition of associative free functioning identify sessions where the relationship between the patient and the psychoanalyst denotes a positively perceived flow of deep thoughts and understandings. On the contrary, the Perturbating factor correlates negatively with three SEQ factors (Depth, Smoothness, and Positivity). This element emphasizes the innovative dimension of the instrument. The SEQ aims to capture the good/bad quality of the session, while FASS emphasizes how a less known, more sudden trend is a source of interesting changes in analytic work. The novelty of the FASS results, precisely regarding the Perturbing factor, is associated with a clinician's perception of a more complex and unexpected session. Thus, a dual functioning of the associative process seems to emerge: one that is more linear, with connections in the patient's and analyst's minds, and one that is more disruptive, pushing the analyst and the patient to rethink what happened. These results are confirmed by the correlations between the analyst's clinical notes and the two factors of the FASS. The Associativity factor correlates negatively with the number of words used by the clinician. This result appears to indicate that, when the associative flow is effective, the clinician observed the need to narrate and write about the incident. However, the Perturbation factor correlates negatively with symbolization (IWRAD and IWRAD_IWRRL). This indicates how high perturbation denotes a clinical account that is more adherent to the facts of the session and is less metaphorical. Table 4 contains examples of clinical notes.

**Table 4:** Examples.

High Perturbing and Low Associativity scores:
He is a patient who has obsessive traits and tends to use intellectualization. It is not easy to go through the associative way, and here and there, strongly controlled partial associative movements are glimpsed. In today's session, an aspect that was touched on in the past comes out. He dramatically narrates of his holding fecal and fecal incontinene issues that he faced until the age of 11 years. Hence, a description of difficult socialization, a continuous search for solitude in which daydreams prevailed, the desire to bring his peers closer, and also a desire for a command that always put him in crisis with respect to the rest of the group, in the end, marginalized him. The prevailing theme is the comparison with his parents: the patient is the father of 2 kids of latency age, and often in sessions, he talks about his children and the comparison with his parents becomes natural. It is difficult to feel free in the session, and I often find myself distracted, as if I lose my thread even when, like today, he tells about interesting life material that arises in the session and that is not already prepared previously; even today, in some passages, I was bored; however, compared to other sessions, this was not bad!
**High Perturbing and High Associativity scores:**
The patient begins by saying that, one night during the weekend, he had two dreams. In the first, he witnessed a scene in which an adult man dressed in the dark was trying to seduce a young boy. He tells it in a calm tone, saying that he didn't wake up too distressed and kept repeating how much he feels better and changed and that he no longer happens to linger in melancholy in those memories. In the same tone, he tells the second dream and I realize I'm missing details. There are guys in the “Tiger” shop, where they buy fun things. I name the lightness of a situation, as opposed to the tiger. The patient remembers the toys he played with as a child and was happy. “Before it happened.” He realizes he never told me about it. Let's talk about games freely. Another dream that comes to mind is that he had had the night before the session. He tenderly held in his arms his partner's newly born granddaughter, whom they have never seen. It is a very tender scene that contrasts with that of the first dream…We believe that something good can be born and it seems to me that this thought has a less defensive flavor than the “all right, I'm not distressed” as at the beginning.

## 5. Conclusion

This study discussed a newly validated tool in the psychoanalytic field. Starting with the concept of the “fundamental rule,” we were able to expand the construct to various more classical and contemporary definitions of the theory of free association. The important result achieved is that the two hypothesized factors were not only confirmed, but their characteristic independence allows us to state that different phenomena characterize clinical functioning in the analytic exchange. An associative functioning is more consistent with early definitions of the psychoanalytic models, while a second factor that highlights less predictable elements in the analytic relationship relies on theories that are more modern. This second element complements and enhances Freud's functioning hypothesis. By enabling the integration of many theoretical viewpoints, coherent with Ogden's (2022) integration of the epistemic to an ontogenic perspective, we could conclude that this instrument bridges various psychoanalytic theoretical models and reconnects to the receptive position of the analyst, described by Freud as “telephone waves.” This position of broad listening and of receiving the patient's free-associative communications in multiple modes allows for overcoming the logic of discourse and encourages exploration under the surface of speech.

In other words, this tool cuts across different psychoanalytic theoretical models by allowing integration between different theoretical perspectives.

## 6. Limitations

There are many limitations to the study in spite of the relevant sample size that involved most psychoanalysts in Italy. This study should be extended to other cultural and linguistic contexts to confirm the results. Furthermore, the application of the instrument should be extended to other models of psychotherapy, other dimensions of settings, and, in comparison, with patient report instruments to verify the correspondence in the therapeutic relationship.

## Data availability statement

The raw data supporting the conclusions of this article will be made available by the authors, without undue reservation.

## Ethics statement

The studies involving human participants were reviewed and approved by Ethics Committee of the Department of Clinical, Dynamic Psychology and Health studies (Prot. n. 0001104 del 16/07/2021). The patients/participants provided their written informed consent to participate in this study.

## Author contributions

RM, AM, TB, and AF contributed to the conception and design of the study. CD and RM organized the database. LC performed the statistical analysis. RM, AM, and CD wrote the first draft of the manuscript. TB, AF, CC, BG, and AS wrote sections of the manuscript. All authors contributed to the manuscript revision, read, and approved the submitted version.
